# New linear antiplasmodial peptides related to angiotensin II

**DOI:** 10.1186/s12936-015-0974-y

**Published:** 2015-11-04

**Authors:** Adriana Farias Silva, Marcelo Der Torossian Torres, Leandro de Souza Silva, Flávio Lopes Alves, Ana Acácia de Sá Pinheiro, Antonio Miranda, Margareth Lara Capurro, Vani Xavier Oliveira

**Affiliations:** Centro de Ciências Naturais e Humanas, Universidade Federal do ABC, Rua Santa Adélia, 166, Santo André, SP 09210-170 Brazil; Instituto de Biofísica Carlos Chagas, Universidade Federal do Rio de Janeiro, Rio de Janeiro, RJ, Brazil; Departamento de Biofísica, Universidade Federal de São Paulo, São Paulo, SP Brazil; Departamento de Parasitologia, Instituto de Ciências Biomédicas II, Universidade de São Paulo, São Paulo, SP Brazil

**Keywords:** Angiotensin II, Antiplasmodial, Peptides, *Plasmodium falciparum*, *Plasmodium gallinaceum*

## Abstract

**Background:**

Antiplasmodial activities of angiotensin II and its analogues have been extensively investigated in *Plasmodium gallinaceum* and *Plasmodium falciparum* parasite species. Due to its vasoconstrictor property angiotensin II cannot be used as an anti-malarial drug.

**Methods:**

This work presents the solid-phase syntheses and liquid chromatography and mass spectrometry characterization of ten linear peptides related to angiotensin II against mature *P. gallinaceum* sporozoites and erythrocyte invasion by *P. falciparum*. Conformational analyses were performed by circular dichroism. IC_50_ assays were performed to identify the ideal concentration used on the biological tests and haemolytical erythrocytic assays were made to verify the viability of the biological experiments. The contractile responses of the analogues were made to evaluate if they are promising candidates to be applied as antiplasmodial drugs.

**Results:**

The results indicate two short-peptides constituted by hydrophobic residues (5 and 6) with antiplasmodial activity in these models, 89 and 94 % of biological activity against *P. gallinaceum* sporozoite, respectively, and around 50 % of activity against *P. falciparum*. Circular dichroism spectra suggested that all the peptides adopted β-turn conformation in different solutions, except peptide 3. Besides the biological assays IC_50_, the haemolysis assays and contractile response activities were applied for peptides 5 and 6, which did not present expressive results.

**Conclusions:**

The hydrophobic portion and the arginine, tyrosine, proline, and phenylalanine, when present on peptide primary sequence, tend to increase the antiplasmodial activity. This class of peptides can be explored, as anti-malarial drugs, after in vivo model tests.

**Graphical abstract:**
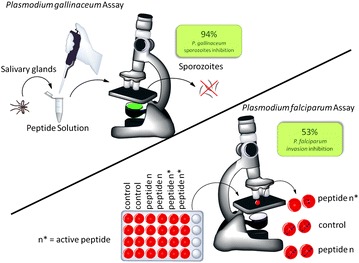
The most active peptide presented 94 % activity on *P. gallinaceum* sporozoites and 53 % inhibited *P. falciparum* ring forms invasion

**Electronic supplementary material:**

The online version of this article (doi:10.1186/s12936-015-0974-y) contains supplementary material, which is available to authorized users.

## Background

Antiplasmodial activities of angiotensin II (Ang II) and its analogues have been extensively investigated [1–6]. Due to its vasoconstrictor property, Ang II cannot be used as an anti-malarial drug regardless of its antiplasmodial activity (in vitro assays) in *Plasmodium gallinaceum* sporozoites [1] and *Plasmodium falciparum* [6].

It is known that protein or peptide-folding mechanism involves a complex bunch of elementary reactions and the different energies associated with positioning of the different amino acid residues near or far from each other or from solvent enable some structures to be more stable than others [7, 8]. Likewise, protein or peptide can be made synthetically and it is a tool for different kind of studies [8, 9].

Chamlian et al. [2] studied some lactam bridged Ang II analogues, which presented Asp and Lys insertion in order to restrict the peptide. They observed that VC-12 (Asp-Arg-Val-Tyr-Ile-Asp-His-Lys-Pro-Phe) and VC-26 (Asp-Arg-Val-Tyr-Asp-Ile-His-Lys-Pro-Phe) analogues showed relevant antiplasmodial activity against *P. gallinaceum* sporozoite, 87 and 73 %, respectively.

In order to understand the role of each amino acid and its side chain, Silva et al. [3] and Ferreira et al. [4] proposed different modifications in the Ang II molecule. They replaced each amino acid by Ala [4] or deleted the residues of the native Ang II molecule [4]. The biological activities of the analogues Asp-Arg-Val-Tyr-Ala-His-Pro-Phe and Asp-Arg-Val-Tyr-Ile-Ala-Pro-Phe on the *P. gallinaceum* sporozoites were equipotent to native Ang II [4], 75 and 79 % of activity, respectively. The most active analogues studied by Ferreira et al. presented biological activities about 50 % (analogues Asp-Arg-Val-Tyr-Ile-His-Pro and Arg-Val-Tyr-Ile-His-Pro-Phe). They synthesized three short peptides (Val-Tyr-Ile-His-Pro-Phe; Val-Tyr-Ile-His-Pro and Ile-His-Pro-Phe) to verify the importance of hydrophobic cluster studied by Tzakos [10] and Fermandjian [11, 12]. The peptides presented antiplasmodial activities about 80 %. These studies were important to understand the position of each amino acid side chain and intra/intermolecular interactions, which play an important role in the native sequence, and that the hydrophobic cluster have significant influence on both cases.

In this work, new linear peptides and Ang II analogues were synthesized and tested in vitro in order to find a short bioactive peptide as well as to verify the hydrophobic cluster’s influence on parasite-membrane interaction on both *P. gallinaceum* and *P. falciparum* parasite species, as previously mentioned.

## Methods

### Peptide synthesis, purification and characterization

The peptides were synthesized using a common protocol for manual solid-phase synthesis; the Fmoc [13] strategies were applied and Wang resins [14] (aapptec, USA) with a substitution degree of 0.55 mmol g^−1^ were used. Amino acids (Novabiochem, USA) deprotection steps were carried out by treatment with 4-MePip in DMF (40 min). Couplings were carried out using a 2.5-fold excess of DIC/HOBt in DCM/DMF (1:1, v/v) and were monitored using the Kaiser ninhydrin test [15]. Dry-protected peptidyl-resin was exposed to TFA/H_2_O/anisole (95:2.5:2.5, v/v/v) for 2 hours, at room temperature. All crude peptides were precipitated with anhydrous diethyl ether, separated from the ether-soluble reaction components by filtration, extracted from the resin with 60 % ACN in water and lyophilized.

The crude lyophilized peptides were then purified by preparative RP-HPLC in 0.1 % TFA/60 % ACN in water on a Waters Associates system (Delta Prep 600). The peptides were loaded onto a Phenomenex C_18_ (21.2 × 250 mm, 15 µm particle size, 300 Å pore size) column at a flow rate of 10.0 mL min^−1^ and eluted using a linear gradient (slope 0.33 % B/min) of TFA/ACN with detection at 220 nm. Selected fractions containing the purified peptides were pooled and lyophilized.

Purified peptides were characterized by LC/ESI–MS. LC/ESI–MS data were obtained on a Micromass instrument (model ZMD) coupled to a Waters Alliance system (model 2690) using a Phenomenex Gemini C_18_ column (2.0 × 150 mm, 3.0 µm particle size, 110 Å pore size). Solvent A was 0.1 % TFA in water, and solvent B was 60 % ACN in solvent A. The gradient was 5–95 % B for 30 min, and peptides were detected at 220 nm. Mass measurements were performed in a positive mode with the following conditions: mass range between 500 and 2000 *m*/*z*, nitrogen gas flow rate at 4.1 L h^−1^, capillary voltage at 2.3 kV, cone voltage at 32 V, extractor voltage at 8 V, source heater set at 100 °C, solvent heater set at 400 °C, ion of at 1.0 V and a multiplier at 800 V.

### Circular dichroism

Far-UV (195–260 nm) CD spectra were recorded at 20 °C using a 0.5-mm path-length quartz cell in a Jasco J815 spectropolarimeter (Tokyo, Japan). All spectra were recorded after an accumulation of four runs. The scan rate was 50 nm min^−1^ for all measurements with bandwidths of 0.5 nm. All peptides were measured in the following four solutions: 15 mmol L^−1^ PBS (pH = 7.4), 10 mmol L^−1^ SDS, 50 % TFE in PBS, and 50 % MeOH in PBS. The peptide concentration was 10^−4^ mol L^−1^. The CD spectra for the SDS, TFE and MeOH solutions were subtracted, and a Fourier transform filter (FFT) was applied to minimize background effects.

### Bioassays

#### Mosquito rearing and maintenance of the parasite life cycle

The RED strain of *Aedes aegypti* is highly susceptible to *P. gallinaceum* [16] and was used in all experiments. Mosquitoes were reared using standard laboratory procedures [17]. An aliquot of frozen chicken blood infected with the *P. gallinaceum* strain 8A was obtained from A Krettli (René Rachou Institute of Research, FIOCRUZ, MG, Brazil). This sample was used to inoculate and establish initial infections in chickens. All subsequent infections of chickens and mosquitoes were accomplished by feeding the mosquitoes on the chickens.

#### Effect of the peptides on salivary gland-derived *Plasmodium gallinaceum* sporozoites

Nine-thousand *P. gallinaceum* mature sporozoites were recovered from the salivary glands of *Ae. aegypti* and incubated in 50 μL of the PBS solution, with 40 μmol L^−1^ digitonin (positive control), 60 μmol L^−1^ peptides or negative controls, at 37 °C for 1 h. Cell membrane integrity was then monitored using a Carl Zeiss inverted fluorescence microscope (model Observer Axio Vision A.1) coupled to an image capture Zeiss AxioCam HR digital camera (1300 × 1030 pixels resolution and 8-bit quantization) after addition of 1 μL of the propidium iodide aqueous solution (200 μmol L^−1^) in 5 μL of total solution volume. Images were obtained using a 40× objective lens and a green filter effect in red. The spectral range was set with the excitation at 538 nm within the visible spectrum in order to produce orange-red fluorescence centered at 619 nm, which was processed using the Axio 4.7 software.

#### *Plasmodium falciparum* culture in vitro

The erythrocytic cycle of *Plasmodiumfalciparum* was maintained in vitro by culturing W2 strains in 1640 medium, supplemented with A-type human blood and serum, as described in Saraiva et al. [6]. The parasite first samples were gently donated by Dr. Mariano Zalis from the Laboratory of Infectiology and Parasitology, Hospital Universitário Clementino Fraga Filho, Universidade Federal do Rio de Janeiro, Rio de Janeiro, Brazil.

#### Erythrocytic invasion by *Plasmodium falciparum*

Sorbitol solution (5 %) was used to synchronize parasite cultures into the ring stage, according to Lambros and Vanderberg [18]. The resistant ring-forms are subsequently maintained in culture as described above. Erythrocytes infected by parasite mature forms at 2–3 % parasitaemia and 5 % haematocrit were kept in the presence or absence of 10^−8^ mol L^−1^ synthetic peptides, for 24 h. Invasion is assessed by the appearance of new ring forms. The percentage of invasion was determined by optical microscopy (1000× magnification). It represents the total number of cells infected by rings in 100 erythrocytes in at least ten random microscopic fields. The invasion assay was carried out using two different cell suspension and generated equivalent results

#### IC_50_ value determination

A dose response was carried out using increasing concentrations (10^−4^, 10^−6^, 10^−8^, 10^−9^, 10^−10^, 10^−12^, 10^−14^ mol L^−1^) of each peptide, for 24 h, in order to determine the IC_50_ for peptides 5 and 6 in the suppression of erythrocytic invasion by *P. falciparum*. The IC_50_ values were calculated by using GraphPad Prism Software version 5.00 for Windows (GraphPad, San Diego, California, USA). Parameters: Nonlinear Regression; log (inhibitor) vs. response equation was chosen at least square (ordinary) fit method was applied.

#### Haemolytic effect of peptides

The haemolytic effect of analogs 5 and 6 was determined in uninfected erythrocytes. Cells were maintained in the same culture conditions in the presence of 10^−8^ mol L^−1^ peptides, for 24 h. Erythrocytes were centrifuged at 900*g*/8 min and free haemoglobin in the supernatant was assessed by spectrophotometer at 550 nm. Untreated cells were used as negative control to discount non-specific haemolysis. The haemolytic activity was expressed as percentage of the control. This positive control (100 % haemolysis) was prepared by treating the erythrocytes with distilled water.

#### Minimum haemolytic concentration (MHC)

The MHC of the peptides were assessed through the measurement of haemoglobin released by non-infected human erythrocytes in the presence of increasing concentrations of the compounds in the same culture conditions of the invasion assay. As described by Jin-Jiang [19], MHC is the highest peptide concentration that causes no detectable release of haemoglobin.

#### Minimum inhibitory concentration (MIC)

A dose–response assay was carried out in similar conditions of the invasion assay for MIC determination. MIC was determined as the lowest peptide concentration able to inhibit parasite invasion. It is used to evaluate the compound activity [20].

#### Contractile response assays

The bioassay experiment was carried out in duplicate and generated equivalent results. Experiments were carried out with C57BL/6 J mice from the Centro de Desenvolvimento de Modelos Experimentais da Universidade Federal de São Paulo (CEDEME-UNIFESP). Stomach fundus was isolated from each mouse, divided in two strips along the longitudinal muscle, and mounted into 5-ml organ baths containing modified Krebs–Ringer solution [144 mmol L^−1^ NaCl, 5 mmol L^−1^ KCl, 1.1 mmol L^−1^ MgSO_4_, 25 mmol L^−1^ NaHCO_3_, 1.1 mmol L^−1^ NaH_2_PO_4_, 1.25 mmol L^−1^ CaCl_2_, and 5.5 mmol L^−1^ glucose at 37 °C (pH 7.4)] and were continuously carboxygenated (95 % O_2_/5 % CO_2_) [21]. Contractile responses to the stimulations with Ang II and analogue 5 and analogue 6 (10^−8^ mol L^−1^) were measured with a TRI201 tension transducer (PanLab) through an amplifier (Powerlab 4/30). Data were collected through Labchart Pro V7 software. The resting tension was maintained at 0.5 g and the tissues were left to equilibrate for 90 min. The bath solution was frequently changed. The maximal effect was obtained by comparing contractile responses induced by carbachol (10^−8^ mol L^−1^).

### Statistical analysis

The experiment was performed in independent cell suspensions. The data were analysed by one-way analysis of variance using treatments as factors. The significance of the differences was verified by the Bonferroni adjustment. Statistical analysis was performed using absolute values; the data are expressed as the mean ± standard deviation: n = 2. All of them were considered statistically significant compared with the control value (or water distilled in erythrocytic haemolysis assay), if *p* < 0.05. GraphPad Prism version 5.00 for Windows was used (GraphPad Software, San Diego, California, USA).

### Ethics statement

#### *Plasmodium falciparum* and erythrocyte haemolysis assays

The collection of human blood samples for this study was conducted according to the protocols and approved by the Research Ethics Committee of the Hospital Universitário Clementino Fraga Filho at the Universidade Federal do Rio de Janeiro (Permit Number 074/10).

#### *Plasmodium gallinaceum*

The collection of *Gallus gallus domesticus* blood samples for this study was conducted according to the current guidelines for the care and use of laboratory animals as well as the ethical guidelines for investigations, and these experiments were preapproved by the Animal Care Committee of the Universidade de São Paulo, number 133.

#### Contractile response assay

The pharmacological experiment followed the current guidelines for the care and use of laboratory animals as well as the ethical guidelines for investigations, and these experiments were preapproved by the Animal Care Committee of the Universidade Federal de São Paulo, number 2013/479357.

## Results

### Peptide synthesis and characterization

The peptides were synthesized in order to evaluate their influence as antiplasmodial compound. These analogues were purified and characterized as described in Methods, resulting in a chromatographic purity higher than 95 % in all cases. Mass characterization by LC/MS–ESI(+) confirmed the chemical identity of peptides were in agreement with the expected theoretical values (Table 1).

**Table 1:** Purity of peptides determined by LC/MS

Entry	Name	Sequence	HPLC purity^a^ (%)	Calcd mass^b^ (g mol^−1^)	Obsd mass^b^ (g mol^−1^)
1	[Ile^6^,His^5^]-AII	DRVYHIPF	98	1045.5	1046
2	des-Ile^5^,His^6^-AII	DRVYPF	98	795.4	796
3	des-Asp^1^,Val^3^,Ile^5^,His^6^-AII	RYPF	99	581.3	582
4	[Ile^4^,His^3^]-des-Asp^1^,Val^3^-AII	RYHIPF	98	831.4	833
5	des-Asp^1^,Arg^2^,Val^3^,Ile^5^-AII	YHPF	99	562.2	563
6	des-Asp^1^,Arg^2^,Tyr^4^,His^6^-AII	VIPF	99	474.3	475
7	[2-Nal^1^]- des-Asp^1^,Arg^2^,Tyr^4^,His^6^-AII	(2-Nal)-IPF	97	572.3	574
8	[2-Nal^2^]- des-Asp^1^,Arg^2^,Tyr^4^,His^6^-AII	V-(2-Nal)-PF	99	558.3	559
9	[2-Nal^3^]- des-Asp^1^,Arg^2^,Tyr^4^,His^6^-AII	VI-(2-Nal)-F	97	574.3	575
10	[2-Nal^4^]- des-Asp^1^,Arg^2^,Tyr^4^,His^6^-AII	VIP-(2-Nal)	98	523.3	524

^a^ HPLC profiles were obtained under the following conditions: Column Supelcosil C_18_ (4.6 × 150 mm), 60 Å, 5 μm; Solvent System: A (0.1 % TFA/H_2_O) and B (0.1 % TFA in 60 % ACN/H_2_O); Gradient: 5–95 % B in 30 min; Flow: 1.0 mL/min; λ = 220 nm; Injection Volume: 50 μL and sample concentration: 1.0 mg/mL

^b^The masses were determined by LC/ESI–MS using a Micromass instrument (model ZMD) coupled to a Waters Alliance (model 2690) system. Mass measurements were performed in a positive mode with the following parameters: mass range between 300 and 2000 m/z; nitrogen gas flow: 4.1 L/h; capillary: 2.3 kV; cone voltage: 32 V; extractor: 8 V; source heater: 100 °C; solvent heater: 400 °C; ion energy: 1.0 V and multiplier: 800 V

### Circular dichroism

**Fig. 1:**
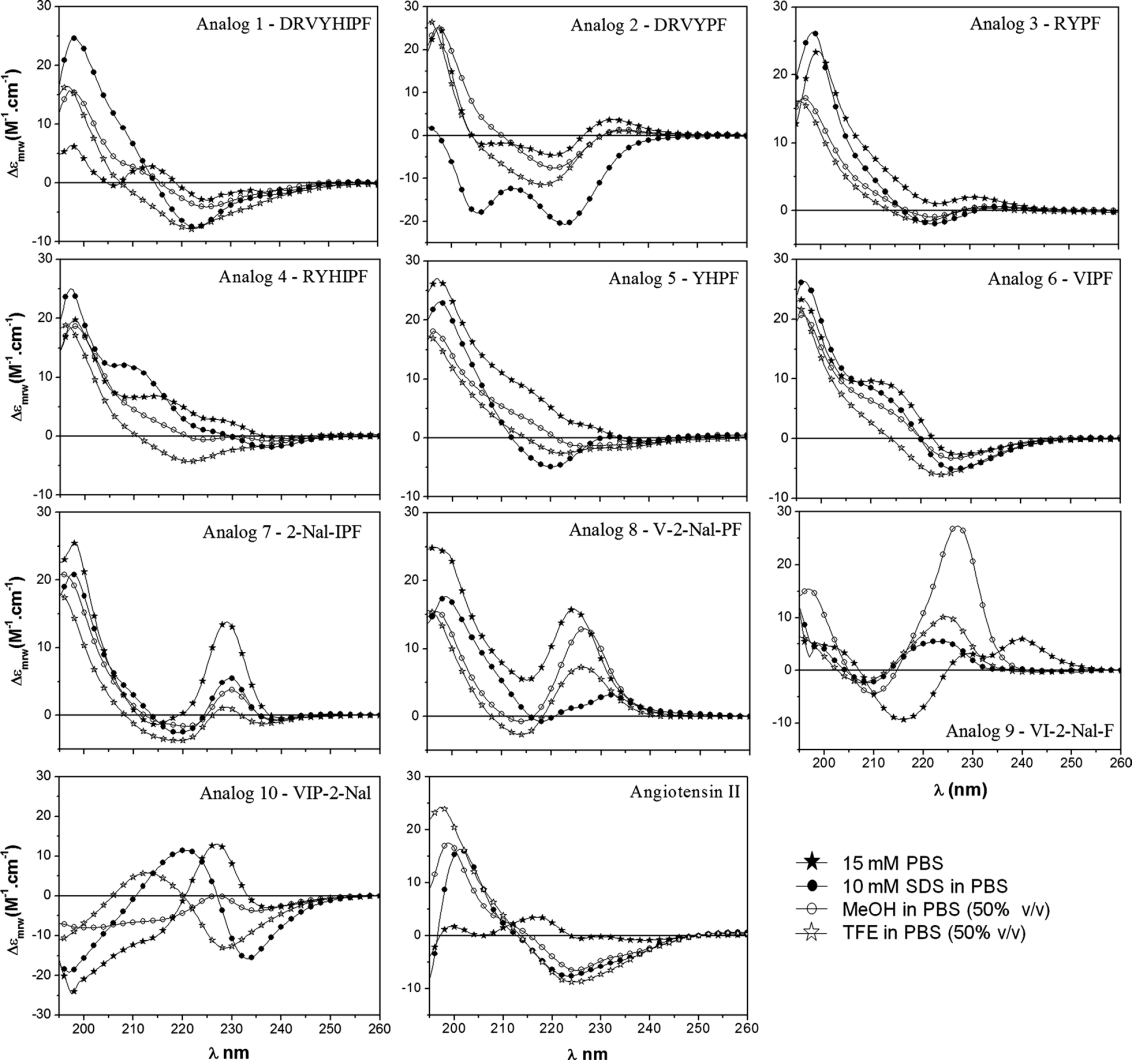
CD spectra were recorded after four accumulations at 20 °C using a 0.5-mm path-length quartz cell between 260 nm and 195 nm at 50 nm/min with a band width of 0.5 nm. All peptides were analysed in the following four solutions: 15 mM PBS, 10 mM SDS/PBS, 50 % TFE/PBS, and 50 % MeOH/PBS. The peptide concentration was approximately 10^−4^ mol L^−1^

CD spectroscopy is a method that has been used to investigate proteins, polypeptides and peptides secondary structures and tendencies to structure [22]. It also has been used to verify conformational peptides changes that could occur during peptide-membrane interactions [23, 24]. Therefore, to understand the peptides conformational changes, inter- or intramolecular interactions, the CD analyses were performed in specific solvents. SDS was used to simulate peptide-membrane interactions, simulating natural biological environments [25]; TFE induce an α-helix formation and stabilize the secondary structure [26]; MeOH promotes β-turns conformation The results suggest that all peptides, except peptide 3, tend to adopt β-turns conformation (Fig. 1) [27].

### Effect of te peptides on salivary gland-derived *Plasmodium gallinaceum* sporozoites

**Fig. 2:**
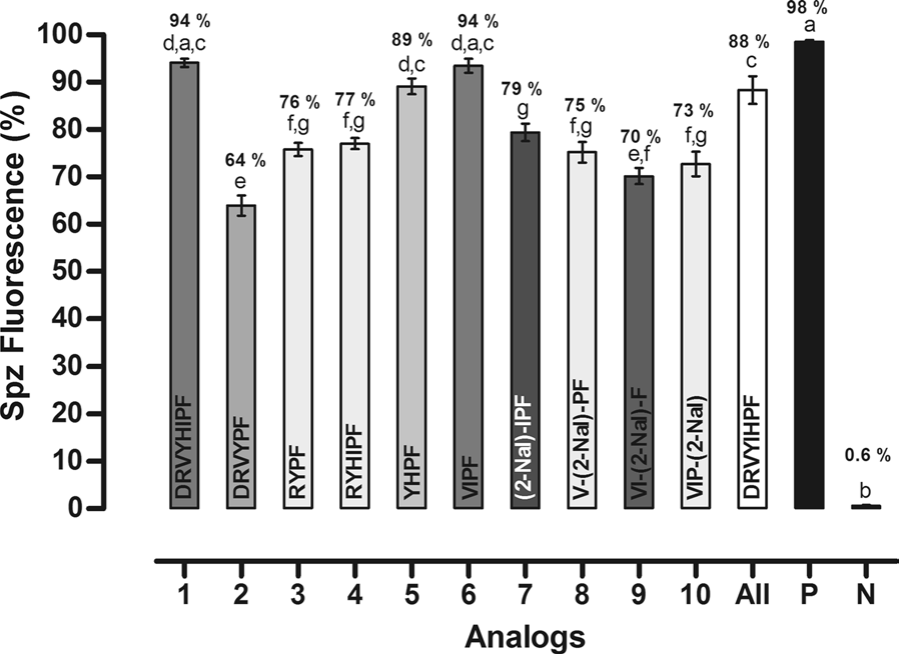
Effects of peptides on membrane permeability expressed as the per cent of fluorescent mature sporozoites (mean ± standard deviation, n = 9). *Letters* indicate those results not significantly different from each other at the p < 0.05 level. Positive control group (+): digitonin/PBS; negative control group (−): PBS. The most active peptides were 1, 5 and 6 that presented 94, 89 and 94 % antiplasmodial activity, respectively

The effect of the peptides on sporozoites was determined in vitro by fluorescence microscopy after one hour of incubation of the parasites with each peptide in the presence of propidium iodide (see Additional file 1). The results are reported as the per cent of fluorescent sporozoites. The analyses were carried out in triplicate using three different mosquito batches and a total of nine experiments were performed (n = 9). The effects of the peptides on *P. gallinaceum* sporozoites in vitro assay presented 64–94 % activity. Data are shown in Fig. 2.

### Effect of the peptides in the *Plasmodium falciparum* erythrocytic cycle

**Fig. 3:**
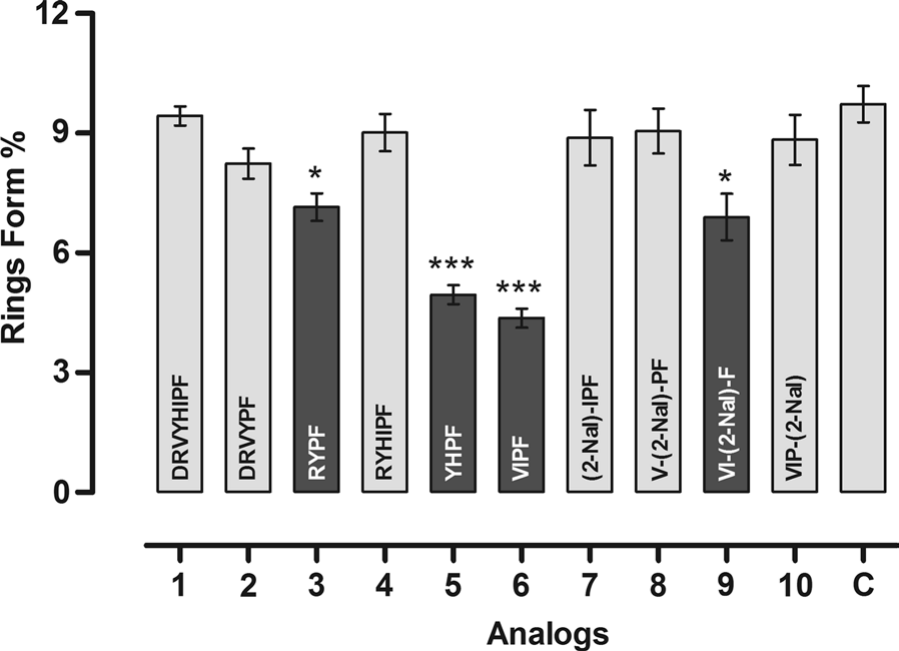
Effect of peptides in the new ring formation. The percentage of rings was determined after 24 h incubation of erythrocyte cultures infected with 2–3 % schizonts in the absence (control) or in the presence of 10^−8^ mol L^−1^ peptides. *Asterisk* Statistically significant compared with control value p < 0.05. *Triple asterisk* Statistically significant compared with control value p < 0.001. *Dark grey* *shading* indicates that the result is statistically significant compared with control (mean ± standard deviation, *n* = 2)

The effect of the peptides in the *P. falciparum* erythrocytic cycle was assayed in vitro against a synchronized culture in the schizont form of the *P. falciparum* maintaining 5 % haematocrit and 2 % parasitaemia. Twenty-four hours after treatment, all peptides reduced new ring formation at 10^−8^ mol L^−1^, which was studied by Saraiva et al. as the ideal concentration for these inhibition assays [6]. The tests were carried out in duplicate and a total of ten experiments were performed (n = 10). Data have been normalized due to difference between controls of each assay. It was observed that four analogues were reduced in the number of rings formed in the blood stage, but only analogues 5 and 6 presented inhibition higher than 50 % compared with control (Fig. 3).

### IC_50_ value determination

**Fig. 4:**
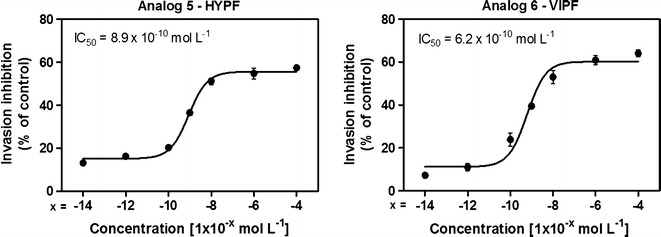
IC_50_ value for each assay. Peptides 5 and 6 were diluted in seven concentrations [(10^−4^; 10^−6^; 10^−8^; 10^−9^; 10^−10^; 10^−12^; 10^−14^) mol L^−1^] giving 7–65 % inhibition. Data have been normalized due difference between control of each assay. The IC_50_ data were analysed by GraphPad Prism analysis. Parameters: non-linear regression; log (inhibitor) vs response equation was chosen and least square (ordinary) fit method was applied (mean ± standard deviation, *n* = 2)

The IC_50_ value represents the peptide concentration producing a 50 % reduction in the number of *P. falciparum* rings formed compared to peptide-free control cultures. The concentration of each peptide required to inhibit the rings form by 50 % (IC_50_) was determined by testing seven concentrations giving 7–65 % inhibition and then interpolating IC_50_ by non-linear regression analysis as previously cited. Data are shown in Fig. 4.

### Haemolytic effect of peptides

**Fig. 5:**
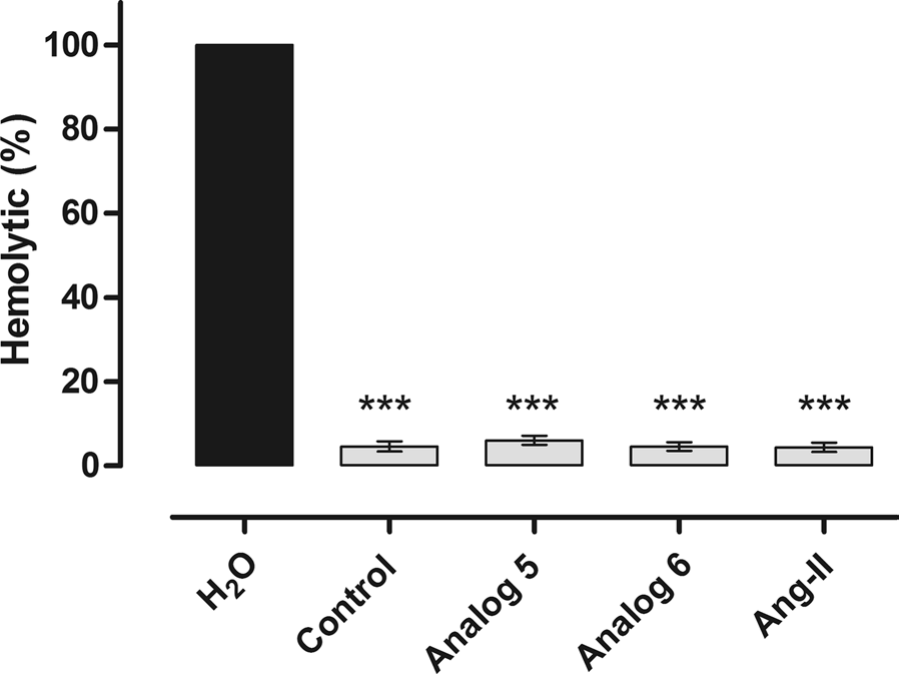
Haemolytic assay of human red blood cells were treated with distilled water; uninfected erythrocytes were kept in the same conditions used in the invasion assay with a control and each peptides tested (10^−8^ mol L^−1^), at 37 °C for 24 h. After incubation, the supernatant was collected, clarified at 900 g/8 min and the haemoglobin content was detected in a spectrophotometer at 530 nm. *Triple asterisk* statistically significant compared with distilled water value p < 0.05. *Light grey shading* indicates that the result is not statistically significant compared with control (mean ± standard deviation, *n* = 3)

The effects of the Ang II, peptides 5 and 6 in the erythrocyte human cells to define the haemolysis were seeded in a 96-well plate in the absence (control) or presence of 10^−8^ mol L^−1^ of the analogues, as already described. The peptides did not present hemolytic effects. Data are shown in Fig. 5.

### Therapeutic index (MHC/MIC Ratio)

The therapeutic index is a parameter used to represent the specificity of antimicrobial compounds. It was calculated by the ratio of MHC and MIC. Higher values in therapeutic index represent greater antimicrobial specificity. The minimum haemolytic activity for peptides 5 and 6 was calculated and compared to Ang II. Both of them had the same MIC value, however since MHC values were not the same. It resulted in different specificity for each peptide represented by MHC/MIC ratio, which was higher in peptide 5 (peptide: 10^9^; peptide 6: 10^8^) (Table 2).

**Table 2:** Therapeutic index determination (MHC/MIC ratio)

	MHC [M]	MIC [M]	MHC/MIC
Ang II	10^−5^	10^−12^	10^7^
Peptide 5	10^−5^	10^−14^	10^9^
Peptide 6	10^−6^	10^−14^	10^8^

Therapeutic index was determined for the compounds that had anti-plasmodial activity during the erythrocytic cycle of *P. falciparum*

### Contractile response of peptide 5 and 6

**Fig. 6:**
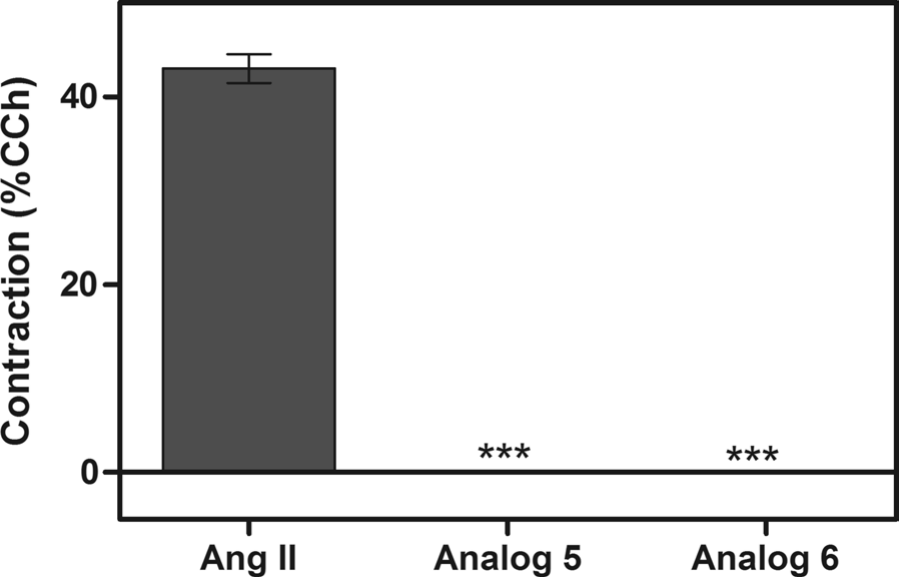
Effects of Ang II, peptides 5 and 6 in contractile responses by muscle tissue incubation compared to carbachol (CCh) activity. *Triple asterisk* statistically significant compared with control value p < 0.05 (mean ± standard deviation, *n* = 2)

Results obtained in the contractile response assays showed that out of the two peptides tested, none presented contractile activity compared to Ang II and carbachol (Fig. 6).

## Discussion

Silva et al. observed that when the Arg, Tyr, Pro, and Phe amino acids residues were replaced by Ala, the antiplasmodial activity of these peptides decay 50 % in comparison to the native Ang II [3]. Ferreira et al. observed that when the same amino acids residues were deleted, the analogues presented similar activity [4]. Moreover, these authors also observed that ultra-short peptides, designed based on hydrophobic C-terminal extremity of Ang II, increase the antiplasmodial activity [4].

This leads to two questions: Are these amino acid residues (Arg, Tyr, Pro, and Phe) relevant on Ang II backbone to maintain the antiplasmodial activity? Does the Ang II hydrophobic cluster exert any influence in the peptide-parasite membrane interaction?

Ang II and several analogues tend to β-conformation at studied solvents [4, 5, 28]. In order to answer these questions there were designed and synthesized six peptides (Table 1, peptides 1–6) in order to evaluate their structuring tendency behaviour in different solvents and their antiplasmodial response.

Firstly, the peptides were tested in vitro on *P. gallinaceum* sporozoite model. Based on Ang II primary sequence (Asp-Arg-Val-Tyr-Ile-His-Pro-Phe), the peptides 1 (Asp-Arg-Val-Tyr-His-Ile-Pro-Phe) and 2 (Asp-Arg-Val-Tyr-Pro-Phe) were designed to verify if the hydrophobic residues exert some influence on parasite membrane. The position of His and Ile amino acid residues in peptide 1 was inverted. This promoted an increase in the antiplasmodial activity (94 %). The same residues (His and Ile) were deleted, in peptide 2. This modification leaded to a decreased antiplasmodial activity of 64 %.

Matsoukas et al. suggested that a π-stacking interaction between His and Phe side chains, on Ang II molecule, is an important aspect of the activation mechanism in its biological activity [29]. Due to changes in residue position, on peptide 1, both the hydrogen bond between phenol group of Tyr with imidazole group of His [30], and van der Waals interaction between Ile and Phe residues [12, 31] could promote the peptide-parasite interaction. It cannot occur in native molecule, because Ile side chain presents a steric hindrance towards Tyr. Besides that, His spatial organization leads to the stability of the Ang II conformation [12].

The deletion of His and Ile residues, as peptide 2, can be responsible for the biological activity decrease. It was observed in CD spectrum (Fig. 1) that peptide 2 tends to adopt an α-helix structuring (i.e., a positive band near 205 nm and two negative bands near 208 nm and 222 nm) in SDS solvent, even for hexapeptides [32, 33], while peptide 1 tends to adopt a β-turns structuring (i.e., a positive absorption near 195 nm and a minimum near 225 nm) also in SDS solvent. (See Additional file 2).

Arrighi et al. studied three different conformations (random coil, α-helix and β-sheet) with murine parasite and suggested that only α-helix structuring did not present efficacy with parasite membrane activity [34]. This information is in accordance with peptide 2, which leads to decreased biological activity. Based on the previous result and considering that the hydrophobic residues are responsible for peptide-parasite membrane interaction, the Ang II derivative (peptide 3, Arg-Tyr-Pro-Phe) was designed to verify if Arg, Tyr, Pro, and Phe residues are relevant for this interaction [4]. The peptide 4 (Arg-Tyr-His-Ile-Pro-Phe) was designed to verify if His and Ile amino acid residues addition at primary sequence of peptide 3 enhance the biological activity (Figs. 1, 2).

The results showed that, in this case, the activity was equipotent (77 and 76 % antiplasmodial activity, respectively). The effect of the cluster formed by Ile and His inverted residues was not significant in biological activity of the peptide 4, because the interactions between Arg and Tyr residues are predominant when compared to the interactions between Tyr and His residues [35].

The Arg and Tyr residues are not adjacent in peptide 1. It probably leads to the influence of the cation-π interactions between Tyr and Arg residue side chains contained in the molecule providing greater stability [36]. This hypothesis can explain the higher activity of peptide 1 than peptide 4 and because peptides 3 and 4 present lower biological activities. CD spectra for both peptides (3 and 4) tend to β-turns conformation.

Silva et al. also suggested that the aromatic or hydrophobic residues can promote peptide-parasite interactions [4]. Thus, peptides 5 (Tyr-His-Pro-Phe) and 6 (Val-Ile-Pro-Phe) were designed based on this data. The bioactivities observed for both peptides were 89 and 94 %, respectively. It leads to the deduction that short-peptides were able to maintain the antiplasmodial activity of this class of molecules.

Cruzeiro-Silva et al., by NMR studies, analysed possible interactions of the PW2, an antimicrobial synthetic peptide (His-Pro-Leu-Lys-Gln-Tyr-Trp–Trp-Arg-Pro-Ser-Ile), simulating several lipophilic environments [37]. In those studies, they suggested the aromatic region comprises the primary sequence Trp–Trp-Arg (a slice of PW2 sequence) that is responsible by anchoring the peptide in the membrane interface and these same regions display some degree of conformational order in solution [37]. This information was relevant to elucidate the decrease of peptide 5 antiplasmodial activity and/or its interaction with the parasite membrane.

Perez-Picaso et al. studied the hydrophobicity of the amino acid residues and associated it with anti-malarial activity [38]. They suggested that among the residues on peptides that tend to act as anti-malarials are: Val, Ile, Pro, and Phe, the same applied to design peptide 6, besides other bulky or hydrophobic residues such as Trp and Arg [38]. It is in accordance with the results obtained in this work. Peptides 5 and 6 tend to adopt β-turn structuring. Peptides that tend to adopt β-turn structures present higher interaction with *P. gallinaceum* sporozoites membrane (>75 %) [4, 5]. The same characteristic was observed here.

In order to find a potent antiplasmodial with 100 % of efficacy, the peptide 6 was modified. Each amino acid residues was replaced by 2-Nal. This was chosen because it has the hydrophobic bulky side chains. The 2-Nal scan library of peptide 6 derivatives was realized, obtaining peptides 7 to 10, Table 1. CD studies were performed (Fig. 1) and their activities against mature *P. gallinaceum* sporozoites (Fig. 2). Meyer et al. studied the substitution of aromatic residues as Pro and Trp by 2-Nal and 1-Nal residues in a β-hairpin peptide [39]. They observed that the molecular geometry was maintained intact when Phe was replaced by 2-Nal residue [39].

It was observed that the substitution in the C-terminal extremity caused a decrease on peptide-parasite interaction (peptides 9 and 10, 70 and 73 %, respectively). The increase of the biological activity was observed by Chamlian et al. [2] in Ang II analogues, when C-terminal extremity is modified with two amino acids insertion (Asp and Lys). Despite the antiplasmodial activity decay of peptides 7 and 8 when compared to peptide 6, CD spectra suggest that in general these two peptides adopt similar conformational tendency in all solvent systems. A positive band in ~200 and ~230 nm and a negative band in ~218 nm indicates that peptides 7 and 8 tend to adopt β-turn structuring [40]. Besides that, a sharp positive band at ~230 nm indicates the presence of naphthyl group, as reported by Ueno et al. [41].

The peptides tend to present antiplasmodial activity when they adopt similar structuring in all solvents studied [4]. It was observed in peptide 1 in comparison to Ang II or peptide 5 when compared to peptide 6. The CD spectra suggested that β-turn conformations are observed for the most bioactive peptides in aqueous organic means with the presence of SDS. The structuring tendency presented by this class of peptides introduced here is in accordance with the results presented here.

Saraiva et al. described studies that aimed to identify the molecular mechanisms induced by Ang II, which are involved in the modulation of *P. falciparum* erythrocytic cycle. The results showed that Ang II had some influence in the parasite ring forms reduction when tested in vitro (47 % at 10^−8^ mol L^−1^) [6]. Torres et al. studied the influence of restrict peptides in *P. falciparum* red blood cells. Three analogues reduced more than 30 % of the parasitaemia and one of them (Cys-Arg-Asp-Cys-Val-Tyr-Ile-His-Pro-Phe) was active in two different parasite species (*P. falciparum* and *P. gallinaceum*) [5]. In order to verify if some peptides have influence in the same stage, all of them were tested in vitro on red blood cells infected with *P. falciparum* species.

Four peptides presented activity in the parasite ring forms reduction between 27 and 53 % (Fig. 3) and this contribution was noticed only on ultra short-peptides containing Val, Ile, Pro, Phe, Tyr, Arg, and/or His residues in their primary sequences. The results presented in this work are also in accordance with Perez-Picaso’s observations [38].

It was observed that two ultra short-peptides had influence at parasitaemia in both models studied (peptides 5 and 6). IC_50_ values of these two peptides were determined by testing seven concentrations resulting in 7–65 % of inhibition (Fig. 4) with 8.9 × 10^−10^ mol L^−1^ (peptide 5) and 6.2 × 10^−10^ mol L^−1^ (peptide 6). Saraiva et al. observed that the Ang II concentration to reduce parasite invasion in a dose-dependent manner with the maximum effect was 10^−8^ mol L^−1^ [6].

In this work, it was observed that the concentration that has the maximum effect was 10^−4^ mol L^−1^ for both peptides (5 and 6) with 57 and 65 % of parasitaemia reduction, respectively. To verify if peptides 5 and 6 have the same effect of the Ang II, they were tested at 10^−8^ mol L^−1^ presenting parasitaemia reductions of 51 and 53 %, respectively (Fig. 4). The results showed that peptides 5 and 6 are equipotent to Ang II [6].

Haemolysis of erythrocytes and cell toxicity of mammalian somatic cells are often thought to be the major parameters of peptide toxicity toward eukaryotic cells [42]. Haemolytic activity is positively correlated with the peptides hydrophobicity [43]. Likewise, it was verified by erythrocytic haemolysis assays that these short peptides did not affect the integrity of the erythrocytes. They presented haemolytic activity under 7 %. It is not statistically significant when compared to control (Fig. 5). Contractile response assays with the most active peptides showed that they did not exhibit significant contractile activity (Fig. 6).

## Conclusions

New peptides related to Ang II were designed, including the most hydrophobic amino acid residues (Val, Ile, Pro, and Phe), aromatic residues (Tyr, His, Pro, and Phe) and residues from the Ang II hydrophobic cluster (Tyr, Ile and His) in an attempt to verify the peptide-parasite interactions. These peptides exhibited antiplasmodial activity in *P. gallinaceum* sporozoite (64–94 %). Three of them presented activity between 89 and 94 %, maybe due to hydrophobic cluster influence, side chain aromatic rings and hydrophobic residues. CD studies suggested that most of the peptides tend to adopt β-turn conformations and this is in accordance with some previous studies [4, 5]. The derivatives of peptide 6 (2-Nal scan) did not show higher activity. The replacement on the N-terminal extremity presented higher antiplasmodial activity than the replacement on the C-terminal extremity. This is also in accordance with some previous studies [2, 4, 5]. All peptides were tested in *P. falciparum* early trophozoites and four presented activity between 27 and 53 %, including a peptide 6 derivative. Ultra short-peptides (peptides 5 and 6) showed some effect in both parasite species. Furthermore, these peptides did not exhibit haemolysis or contractile response activities. IC_50_ values are more promising than the obtained for Ang II. The results presented here suggest that intramolecular interactions lead to tendency in conformations, which are described as important (add reference) to antiplasmodial activity. The hydrophobic portion and Arg, Tyr, Pro, and Phe residues, when present on peptide primary sequence, led to an increase in antiplasmodial activity. After in vivo model tests of this class of peptides, this kind of studies contribute to the development of new chemotherapeutics, which can be explored as anti-malarial drugs.

## Additional files

10.1186/s12936-015-0974-y Fluorescence Microscopy Image. (A) sporozoites in phase (left) and (B) fluorescent microscopy (right).
10.1186/s12936-015-0974-y Peptides deconvolution.

## Abbreviations

Arg: arginine
Ala: alanine
Tyr: tyrosine
Asp: aspartic acid
Val: valine
Ile: isoleucine
His: histidine
Pro: proline
Phe: phenylalanine
Lys: lysine
Trp: triptophan
C: cysteine
2-Nal: 2-naphthylalanine
Ang II: angiotensin II
*P*: *Plasmodium*
CD: circular dichroism
Fmoc: fluorenylmethyloxycarbonyl
MeOH: Methanol
4-MePip: 4-methylpiperidine
DMF: dimethylformamide
DIC: 1,3-diisopropylcarbodiimide
HOBt: *N*′,*N*,*N*-hydroxybenzotriazole
DCM: dichloromethane
TFA: trifluoroacetic acid
ACN: acetronitrile
RP-HPLC: reverse-phase high-performance liquid chromatography
LC/ESI–MS: liquid-chromatography electrospray-ionization mass spectrometry
SDS: sodium dodecyl sulfate
TFE: 2,2,2-trifluoroethanol
PBS: phosphate buffer saline
