# Supplementary material for: New linear antiplasmodial peptides related to angiotensin II
Source: Malar J. 2015 Nov 4;14:433. doi: 10.1186/s12936-015-0974-y (PMC4634797; doi:10.1186/s12936-015-0974-y)
Supplement: Supplementary file 1 — 10.1186/s12936-015-0974-y Fluorescence Microscopy Image. (A) sporozoites in phase (left) and (B) fluorescent microscopy (right). [file 12936_2015_974_MOESM1_ESM.docx]

Additional file 1 – Fluorescence Microscopy – Image

**Figure S1** – Fluorescence Invert Microscopy – After incubate period, propidium idodide was added and the sporozoites were examined by fluorescent microscopy. (A) sporozoites in phase (left) and (B) fluorescent microscopy (right).

Effect of the peptides on salivary gland-derived *Plasmodium gallinaceum* **sporozoites**

As described in Material and Methods:

Nine-thousand *P. gallinaceum* mature sporozoites were recovered from the salivary glands of *Ae. aegypti* and incubated in 50 μL of the PBS solution, with 40 μmol L^–1^ digitonin (positive control), 60 μmol L^–1^ peptides or negative controls, at 37^o^C for one hour. Cell membrane integrity was then monitored using a Carl Zeiss inverted fluorescence microscope (model Observer Axio Vision A.1) coupled to an image capture Zeiss AxioCam HR digital camera (1,300 x 1,030 pixels resolution and 8-bit quantization) after addition of 1 μL of the propidium iodide aqueous solution (200 μmol L^–1^) in 5 μL of total solution volume. Images were obtained using a 40X objective lens and a green filter effect in red. The spectral range was set with the excitation at 538 nm within the visible spectrum in order to produce orange-red fluorescence centered at 619 nm, which was processed using the Axio 4.7 software.
