# Supplementary material for: New linear antiplasmodial peptides related to angiotensin II
Source: Malar J. 2015 Nov 4;14:433. doi: 10.1186/s12936-015-0974-y (PMC4634797; doi:10.1186/s12936-015-0974-y)
Supplement: Supplementary file 2 — 10.1186/s12936-015-0974-y Peptides deconvolution. [file 12936_2015_974_MOESM2_ESM.docx]

Additional file 2 – Peptides Deconvolution

CDNN software. developed by the German Gerald Böhm was used to calculate the peptides deconvolution.

The β-conformation sum indicates that the most of the peptides tend to adopt a β-turn conformation.

Data in percentage (%) of secondary conformation

| Angiotensin II | | | | |
| --- | --- | --- | --- | --- |
|  | PBS | SDS | MeOH | TFE |
| α-helix | 8.3 | 10.6 | 12.4 | 13.9 |
| **Antiparallel** | **8.1** | **17.7** | **15.7** | **15.1** |
| Parallel | 14.7 | 15.3 | 14.8 | 14.3 |
| β-turn | 16.5 | 14.2 | 12.3 | 10.8 |
| **Random Coil** | **52.4** | 42.1 | 44.9 | 46.0 |
| Total | 100 | 100 | 100 | 100 |
|  |  |  |  |  |
|  |  |  |  |  |
| Peptide 1 | | | | |
|  | PBS | SDS | MeOH | TFE |
| α-helix | 9.7 | 9.8 | 9.7 | 9.5 |
| Antiparallel | 16.0 | 15.6 | 15.8 | 13.2 |
| Parallel | 15.1 | 15.1 | 15.2 | 16.7 |
| **β-turn** | **17.3** | **17.1** | **17.2** | **17.1** |
| Random Coil | 41.9 | 42.4 | 42.2 | 43.8 |
| Total | 100 | 100 | 100 | 100 |
|  |  |  |  |  |
|  |  |  |  |  |
| Peptide 2 | | | | |
|  | PBS | SDS | MeOH | TFE |
| **α-helix** | **58.0** | **50.7** | 9.7 | 9.7 |
| Antiparallel | 1.5 | 1.0 | 15.9 | 16.0 |
| Parallel | 1.2 | 1.3 | 15.1 | 15.1 |
| **β-turn** | 1.1 | 0.9 | **17.3** | **17.4** |
| Random Coil | 38.2 | 45.9 | 42.0 | 41.9 |
| Total | 100 | 99 | 100 | 100 |
|  |  |  |  |  |
|  |  |  |  |  |
|  |  |  |  |  |
|  |  |  |  |  |
|  |  |  |  |  |
|  |  |  |  |  |
| Peptide 3 | | | | |
|  | PBS | SDS | MeOH | TFE |
| α-helix | 9.5 | 9.7 | 9.6 | 9.7 |
| Antiparallel | 15.9 | 15.7 | 15.9 | 15.9 |
| Parallel | 15.3 | 15.2 | 15.2 | 15.2 |
| **β-turn** | **17.1** | **17.2** | **17.3** | **17.3** |
| Random Coil | 42.3 | 42.3 | 42.1 | 42.1 |
| Total | 100 | 100 | 100 | 100 |
|  |  |  |  |  |
|  |  |  |  |  |
| Peptide 4 | | | | |
|  | PBS | SDS | MeOH | TFE |
| α-helix | 8.8 | 9.0 | 9.6 | 9.8 |
| Antiparallel | 16.1 | 11.4 | 16.6 | 9.7 |
| Parallel | 13.8 | 14.7 | 12.9 | 15.2 |
| **β-turn** | **19.5** | **22.4** | **17.5** | **23.3** |
| Random Coil | 42.1 | 42.4 | 43.5 | 42.1 |
| Total | 100 | 100 | 100 | 100 |
|  |  |  |  |  |
|  |  |  |  |  |
| Peptide 5 | | | | |
|  | PBS | SDS | MeOH | TFE |
| α-helix | 8.3 | 9.7 | 9.6 | 9.7 |
| Antiparallel | 13.9 | 15.5 | 12.0 | 15.9 |
| Parallel | 13.4 | 14.9 | 12.1 | 15.2 |
| **β-turn** | **20.3** | **19.5** | **25.7** | **17.3** |
| Random Coil | 44.3 | 40.4 | 40.6 | 42.1 |
| Total | 100 | 100 | 100 | 100 |
|  |  |  |  |  |
|  |  |  |  |  |
| Peptide 6 | | | | |
|  | PBS | SDS | MeOH | TFE |
| α-helix | 9.5 | 9.7 | 9.6 | 9.7 |
| Antiparallel | 15.1 | 15.7 | 14.3 | 15.9 |
| Parallel | 13.0 | 15.2 | 12.9 | 15.2 |
| **β-turn** | **21.7** | **17.2** | **20.4** | **17.5** |
| Random Coil | 40.4 | 42.3 | 42.6 | 41.8 |
| Total | 99.6 | 100 | 99.8 | 100 |
|  |  |  |  |  |
|  |  |  |  |  |
|  |  |  |  |  |
|  |  |  |  |  |
| Peptide 7 | | | | |
|  | PBS | SDS | MeOH | TFE |
| α-helix | 7.7 | 8.2 | 9.6 | 9.7 |
| Antiparallel | 16.0 | 15.7 | 15.9 | 15.9 |
| Parallel | 14.3 | 15.2 | 15.2 | 15.2 |
| **β-turn** | **18.2** | **20.3** | **17.3** | **19.6** |
| Random Coil | 42.5 | 40.8 | 42.1 | 39.8 |
| Total | 98.8 | 100 | 100 | 100 |
|  |  |  |  |  |
|  |  |  |  |  |
|  |  |  |  |  |
|  |  |  |  |  |
| Peptide 8 | | | | |
|  | PBS | SDS | MeOH | TFE |
| α-helix | 9.7 | 9.7 | 9.6 | 10.0 |
| Antiparallel | 16.2 | 14.9 | 15.9 | 15.9 |
| Parallel | 15.7 | 15.2 | 15.2 | 14.4 |
| **β-turn** | **18.3** | **17.5** | **17.3** | **20.4** |
| Random Coil | 40.2 | 42.5 | 42.1 | 39.4 |
| Total | 100 | 99.8 | 100 | 100 |
|  |  |  |  |  |
|  |  |  |  |  |
| Peptide 9 | | | | |
|  | PBS | SDS | MeOH | TFE |
| α-helix | 9.6 | 9.7 | 9.6 | 9.7 |
| Antiparallel | 14.3 | 15.7 | 15.9 | 16.6 |
| Parallel | 13.8 | 15.2 | 15.2 | 15.5 |
| **β-turn** | **19.4** | **17.2** | **18.1** | **17.3** |
| Random Coil | 42.6 | 42.2 | 41.1 | 40.7 |
| Total | 99.7 | 100 | 99.9 | 99.8 |
|  |  |  |  |  |
|  |  |  |  |  |
| Peptide 10 | | | | |
|  | PBS | SDS | MeOH | TFE |
| α-helix | 7.9 | 9.7 | 9.6 | 9.7 |
| Antiparallel | 15.9 | 15.7 | 15.9 | 15.9 |
| Parallel | 17.6 | 15.2 | 15.2 | 15.2 |
| **β-turn** | **20.1** | **17.2** | **17.3** | **17.3** |
| Random Coil | 38.4 | 42.1 | 41.8 | 42.0 |
| Total | 99.9 | 99.9 | 99.8 | 100 |
